# Southern Ocean warming and Wilkes Land ice sheet retreat during the mid-Miocene

**DOI:** 10.1038/s41467-017-02609-7

**Published:** 2018-01-22

**Authors:** Francesca Sangiorgi, Peter K. Bijl, Sandra Passchier, Ulrich Salzmann, Stefan Schouten, Robert McKay, Rosemary D. Cody, Jörg Pross, Tina van de Flierdt, Steven M. Bohaty, Richard Levy, Trevor Williams, Carlota Escutia, Henk Brinkhuis

**Affiliations:** 1 Department of Earth Sciences, Marine Palynology and Paleoceanography, Laboratory of Palaeobotany and Palynology, Utrecht University, Heidelberglaan 2, 3584CS Utrecht, The Netherlands; 2 Department of Earth and Environmental Studies, Montclair State University, 1 Normal Ave, Montclair, NJ 07043 USA; 3 Department of Geography and Environmental Sciences, Faculty of Engineering and Environment, Northumbria University, Newcastle upon Tyne, NE1 8ST UK; 4 NIOZ Royal Netherlands Institute of Sea Research, Landsdiep 4, 1797 SZt Horntje, Texel, The Netherlands; 5 Department of Earth Sciences, Organic Geochemistry, Utrecht University, Heidelberglaan 2, 3584CS Utrecht, The Netherlands; 6 Antarctic Research Centre, Victoria University of Wellington, PO Box 600, Wellington, 6140 New Zealand; 7 Paleoenvironmental Dynamics Group, Institute of Earth Sciences, Heidelberg University, Im Neuenheimer Feld 234, 69120 Heidelberg, Germany; 8 Imperial College London, Department of Earth Science and Engineering, South Kensington Campus, Exhibition Road, London, SW7 2AZ UK; 9 Ocean and Earth Science, University of Southampton, National Oceanography Centre Southampton, European Way, Southampton, SO14 3ZH UK; 10 Department of Paleontology, GNS Science, 1 Fairway Drive, Lower Hutt, 5040 New Zealand; 11 International Ocean Discovery Program, Texas A&M University, 1000 Discovery Drive, College Station, TX 77845 USA; 12 Instituto Andaluz de Ciencias de la Tierra, CSIC-University of Granada, Av. de las Palmeras, 4, 18100 Armilla (Granada), Spain

## Abstract

Observations and model experiments highlight the importance of ocean heat in forcing ice sheet retreat during the present and geological past, but past ocean temperature data are virtually missing in ice sheet proximal locations. Here we document paleoceanographic conditions and the (in)stability of the Wilkes Land subglacial basin (East Antarctica) during the mid-Miocene (~17–13.4 million years ago) by studying sediment cores from offshore Adélie Coast. Inland retreat of the ice sheet, temperate vegetation, and warm oligotrophic waters characterise the mid-Miocene Climatic Optimum (MCO; 17–14.8 Ma). After the MCO, expansion of a marine-based ice sheet occurs, but remains sensitive to melting upon episodic warm water incursions. Our results suggest that the mid-Miocene latitudinal temperature gradient across the Southern Ocean never resembled that of the present day. We demonstrate that a strong coupling of oceanic climate and Antarctic continental conditions existed and that the East Antarctic subglacial basins were highly sensitive to ocean warming.

## Abstract (web summary)

Ocean heat is important in forcing ice sheet retreat, yet past ocean temperature data from proximal ice sheet locations are sparse. Here, the authors present temperature reconstructions from the Wilkes Land subglacial basin during the mid-Miocene, and show that warm waters sustained ice sheet retreat 17–14.8 Ma.

## Introduction

Assessment of the stability and dynamics of the Antarctic ice sheet in a changing climate is fundamental given its role in the climate system including global sea-level change. Observational data and modelling studies suggest consistently that influx of warm waters onto the Antarctic continental shelf invigorates ice retreat^1–3^. Satellite monitoring demonstrates that the rate of basal melting (warm ocean melting the marine-terminated ice sheet margins from below) has exceeded that of surface melting (due to radiative forcing on the ice sheet surface)^1,2^. About 74% of the glaciers covering the Wilkes Land sector of East Antarctica have progressively retreated since 2000 ad due to ingression of warm waters, likely as a consequence of a reduction in sea–ice production and changes in ocean stratification^4^. East Antarctica marine-based subglacial basins have the potential of ~14 m sea level rise and are vulnerable to marine ice sheet instability^5^. Cryosphere–-ocean interactions, therefore, play a critical role in the current ice sheet mass imbalance. Observational data^6,7^ rarely cover a period of time longer than few decades^6^, and are therefore insufficient to provide a record of cryosphere (in)stability at adequately varying CO_2_ concentrations and temperatures as those predicted for the near future. Studying ice sheet instability and ice–ocean interactions during past warm geologic episodes, when atmospheric CO_2_ was analogous to present day or higher, can shed light on the long-term stability of continental cryosphere for our future.

For the mid-Miocene epoch, geological records show major variations in Antarctic ice sheet volume, global sea level, ocean temperatures, and marine fauna and flora^8–11^. The Miocene Climatic Optimum (MCO, ~17–15 Ma) represents one of the warmest intervals since the inception of Antarctic glaciation^12^, with atmospheric CO_2_ concentrations as high as 500–600 parts per million by volume (ppmv)^13–17^, analogous to those expected for the end of the century given unabated carbon emissions. Global surface-ocean and deep-sea temperatures were ~3–6 °C and 5–6 °C above present-day values, respectively^9,18^. The termination of the MCO, widely referred to as mid-Miocene Climatic Transition (MCT; ~14.2–13.8 Ma), was characterised by progressive cooling and an expansion of global ice volume^9,10^; it coincided with a CO_2_ decline to close to pre-industrial values (200–300 ppmv^13–17^). To date, the variability in Antarctic ice sheet volume during the Miocene has mostly been inferred from far-field deep-sea oxygen isotope data^11^ and numerical modelling^18^, and attributed to the combined forcing of atmospheric greenhouse-gas concentrations and orbital variations. However, far-field sedimentary records leave ambiguity as to how much ice volume change is involved in deep-sea oxygen isotope variability, while numerical modelling have difficulties replicating the warm polar climates as derived from proxy data. The few available ice-proximal records, such as from the Ross Sea Antarctic Geological Drilling project (ANDRILL)^19–21^, have provided first evidence for a dynamic ice sheet during the Miocene and suggest extensive melting during peak MCO warmth^21^. Modelling experiments^22^ infer substantial ice-mass loss at boundary conditions of atmospheric ~500 CO_2_ ppmv, astronomical configuration favourable for deglaciation and 2 °C of surface-ocean warming. Ice sheet advances onto the continental shelves, with grounded ice extending into the Ross Sea, are simulated at 280 CO_2_ ppmv and a cold orbit. Hence, both the available field data and modelling efforts indicate that the mid-Miocene Antarctic ice sheets were highly sensitive to relatively small changes in atmospheric CO_2_ concentrations^21,22^. Such sensitivity can be triggered by changes in the ocean dynamics, which are complex, include several feedbacks, and have not yet been considered in most model simulations^23^.

**Fig. 1:**
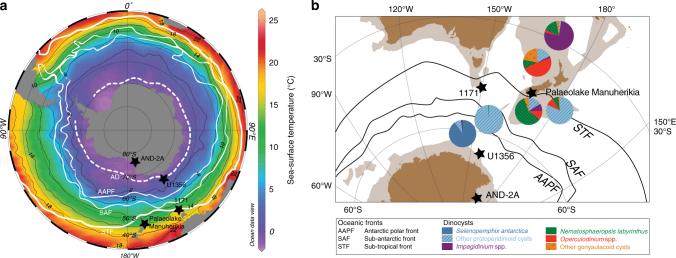
Modern Southern Ocean surface oceanography and dinoflagellate cyst assemblages. **a** Surface-water temperatures of the present-day Southern Ocean during summer (Ocean Data View, https://odv.awi.de/)^90,25^, **b** Dinoflagellate cyst assemblages in surface samples across the southwest Pacific sector of the Southern Ocean as derived by Prebble et al.^32^. Black lines schematically represent oceanic fronts^91^ and black stars indicate the locations of IODP Site U1356 (Wilkes Land)^24^, AND-2A (Ross Sea)^27^, ODP Site 1171 (South Tasman Rise)^9^, and Palaeolake Manuherikia (New Zealand)^28^. STF Subtropical Front, SAF Subantarctic Front, AAPF Antarctic Polar Front, AD Antarctic Divergence. Landmasses are indicated in brown, continental lithosphere in light brown in the location map. Data are derived from Gplates freeware (www.gplates.org; version 1.5.0). We use the plate circuit of ref. ^92^ and the absolute rotation path (based on palaeomagnetism) of the African plate from ref. ^93^ in our reconstructions (see ref. ^46^ for additional information)

Integrated Ocean Drilling Program (IODP) retrieved sediments from Site U1356 (63°18.6138’S, 135°59.9376’E) at the continental rise/abyssal plain boundary, at ~4000 m water depth, ~350 km offshore the Adélie Coast along the Wilkes Land margin, East Antarctica^24^ (Fig. 1). Today, Site U1356 is situated below the Antarctic Divergence, a region of intense upwelling south of the Antarctic Polar Front that is seasonally (~2–3 months per year) sea ice free and characterised by a mean annual sea-surface temperature of ~0 °C (~1–2 °C summer temperature)^25^ (Fig. 1). The Adélie Coast continental shelf is one of the locations where the Antarctic Bottom Waters, the densest water masses of the world ocean, are produced by sea ice formation and the heat loss to the atmosphere^26^. Moving away from the Antarctic margin, the Southern Ocean is characterised by a strong latitudinal surface-temperature gradient and very pronounced oceanographic fronts, the Antarctic (AAPF), subantarctic (SAF) and subtropical (STF) Fronts^25^ (Fig. 1).

The ANDRILL AND-2A record (Ross Sea, 77°45.49’S, 165°16.61’E)^27^, the Wilkes Land Site U1356, and the Ocean Drilling Program (ODP) Site 1171 south of Tasmania (48°30′S, 149°06.69′E)^9^ contain mid-Miocene sediments and are ideally situated along a north–south latitudinal gradient (Fig. 1) to allow investigating the Southern Ocean paleoceanographic condition at times of apparent high continental cryosphere variability^12^. In addition, AND-2A, Site U1356, and Palaeolake Manuherikia (New Zealand)^28^ records (Fig. 1) provide records of continental climate during the mid-Miocene across a latitudinal transect.

Here we document the environmental dynamics characterising both the circum-Antarctic ocean and the Antarctic continent during the early to mid-Miocene based on new palynological (dinoflagellate cysts, pollen, and spores), organic geochemical, and sedimentological data from Wilkes Land Site U1356^24^ in the context of available Miocene data from the Ross Sea AND-2A record^21^ and ODP Site 1171^9^. We demonstrate that marine-based ice sheets are absent or extremely reduced compared to present day during the MCO at the Wilkes Land. Surface waters similar to those found today close to the subtropical front bath this area and sustain ice sheet melting. A much greater ocean temperature gradient between the Ross Sea and the Wilkes Land sites exists compared to the present day, suggesting a different oceanographic structure. Continental conditions at the margin sustain growth of temperate vegetation and soil formation. After the MCO, sea ice occurs and ocean temperatures generally cool at the margin. However, episodic reoccurrence of warm waters destabilises the marine-based ice sheet and the continental cryosphere along the margin is substantially reduced compared to the present day even at pre-industrial atmospheric CO_2_ values. A larger-than-today ocean temperature gradient between the Ross Sea and the Wilkes Land site still exists.

## Results

### Sediment age and sedimentology of Site U1356

Site U1356 was drilled into distal channel levees^24^. The analysed sediment cores are well dated^29^. We herein use novel techniques of constrained optimisation (CONOP)^30^ to further improve the age model (see Methods, Supplementary Figure 1, Supplementary Data 1 and 2). The record spans the time interval from 17 to 10.8 Ma, with a hiatus between 13.4 and 11 Ma, thus comprising the critical intervals of both the MCO and the MCT.

**Fig. 2:**
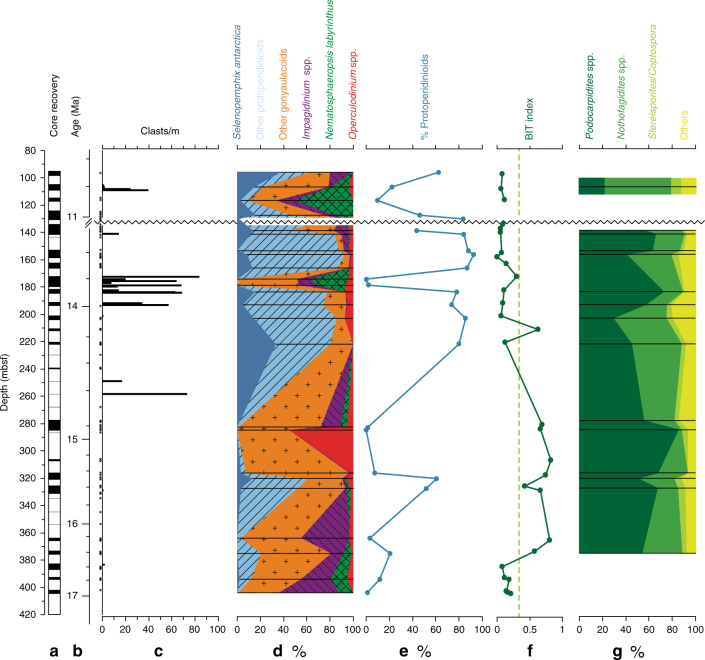
Lithological and palynological results from Hole U1356A. **a** Core recovery; **b** age (Ma) according to the age model described in Supplementary Materials; **c** outsized clast counts (* indicate samples with absence of outsized clasts); **d** synthesis of the marine palynological data; **e** percentages of protoperidinioid dinocysts; **f** organic geochemical biomarker BIT index (dashed line BIT index = 0.3); and **g** terrestrial palynological results

Sediment cores from Site U1356 were described shipboard and post cruise^20^ (Supplementary Figure 2, Supplementary Figure 3, Supplementary Data 3). The MCO interval between 17 and 14.8 Ma (404–275 m below seafloor (mbsf)) is characterised by diatomaceous and cherty mudstones lacking outsized clasts. Metre-scale bioburbated and pinstripe-laminated mudstones with isolated ripple cross-laminated sand interbeds are present. Sporadic distal turbidite beds are indicative of sediment delivery by overbank deposition from active nearby deep-sea channels, which is supported by the seismic stratigraphy indicating a levee depositional setting^24^. Thus, the depositional environment for this interval is interpreted as a distal channel levee setting with subsequent minor reworking by bottom currents of variable strength and bioturbation that do not compromise our paleoenvironmental reconstructions. Ice rafting over the drillsite was probably limited during this time given the paucity of outsized clasts (clasts over 2 mm, see Methods) found in the recovered part of the record (Fig. 2).

In contrast to the MCO interval, sediments between 14.8 and 13.4 Ma (275–133.8 mbsf) are dominated by comparatively higher concentrations of clay and dispersed gravel. Clast-poor diatom-rich beds locally preserve an interlaminated structure of clay-rich diatom ooze and diatom-rich silty clay. Clast-rich mudstones are interbedded with laminated silty claystones. Laminae are predominately sub-mm in thickness, and have sharp upper and lower contacts, suggesting localised reworking of the turbidite levee deposits by bottom currents^31^. Structureless clast-rich mudstones and diamictites are suggestive of iceberg rafted debris delivery and mass transport processes. Abundant clasts occur in three pulses centred at around 14.6, 14.0–13.8, and ~10.8 Ma (260, 180, and 110 mbsf, respectively, Fig. 2). In summary, the depositional environment of Site U1356 is characterised by sediment delivery through hemipelagic settling and overbank turbidity current sedimentation with episodes of bottom current activity and extensive ice rafting. Sediments younger than 11 Ma (above 133.8 mbsf), are separated from underlying units by a hiatus between ~13.4 and 11 Ma and are characterised by preservation of primary turbidite structures including graded silt beds with sharp bases, interbedded with bioturbated muds. Seismic profiles suggest these structures are the result of a migration of the channel levee systems depocentres in the region, which is inferred to be the consequence of ice sheet expansion on the continental shelf after 13.4 Ma^24^.

### Dinoflagellate cysts

To reconstruct relative changes in Miocene seawater temperature, sea ice occurrence, and nutrient availability at Wilkes Land Site U1356, we consider modern ecological affinities of dinoflagellate cysts (dinocysts) in the Pacific sector of the Southern Ocean^32^ (Fig. 1; Supplementary Table 1).

The dinocyst assemblages in the interval between 17 and 14.8 Ma are highly diverse, with temperate phototrophic gonyaulacoid taxa being abundant to dominant (40–98% of the assemblages, Fig. 2, Supplementary Data 4). The taxa found are today mostly abundant in sediments from oceanic marine waters^25^, a setting consistent with the available tectonic reconstructions and core lithology^24^ for the site during the Miocene. A common to abundant component of the assemblages is the genus *Operculodinium*, which today rarely occurs in assemblages south of the Subantarctic Front^33^ (Fig. 1). One *Operculodinium* morphotype (*O*. cf. *centrocarpum*), which occurs in the Ross Sea AND-2A record exclusively in the two warmest pulses of the MCO^19,21^, is here present throughout the record (Supplementary Data 4). Among the *Impagidinium* spp., extant species typical of temperate to tropical (oligotrophic) oceanic environments occur. *I. pallidum* is the only species presently found in Antarctic environments in the vicinity of the polar front, where sea ice occurs, salinity can be seasonally reduced due to ice melting, and seasonality is high^33^. However, this is a species for which a clear tolerance to high temperatures has been demonstrated^33–35^, and its value as sea ice indicator can hence be questioned. Protoperidinioid heterotrophic dinocysts are present, but never abundant except at 15.5 Ma when a single peak (13.5%) of *Selenopemphix antarctica* (Fig. 2) coeval with the sudden appearance of diatom species *Eucampia antarctica*,* Fragilariopsis truncata*, and *Synedropsis cheethamii* ^24^, indicative of colder polar conditions, may point to the occurrence of (seasonal) sea ice. Moreover, the absence or low percentages of reworked dinocysts encountered in this interval (Supplementary Data 4) suggests that deposition occurred in open marine waters.

After ~14.8 Ma, protoperidinioid dinocysts dominate (Fig. 2), indicating high productivity^33^. The encountered taxa include the cosmopolitan *Brigantedinium* spp., *Selenopemphix* spp., and *Lejeunecysta* spp. (Supplementary Data 4). The *Brigantedinium and Lejeunecysta* species found at Site U1356 are the same previously reported from other circum-Antarctic Miocene records^36–38^. *Selenopemphix antarctica* first occurred during the early Oligocene at this site^39^, is almost absent during the MCO, and is commonly present after. This taxon is exclusively known from the present-day Southern Ocean as a dominant component of assemblages from the seasonal sea ice zone south of the Antarctic Polar Front^32^; abundances >20% occur where sea-surface temperatures are <0 °C in winter and spring and up to 10 °C in summer^32,33^ (Fig. 1). Finally, a conspicuous episodic reoccurrence of autotrophic temperate dinocyst species, particularly *Nematosphaeropsis labyrinthus*, is recorded at ~13.8 and ~10.8 Ma (Fig. 2). This species is at present strongly associated with the Subantarctic and Subtropical fronts^32^.

### Pollen and spores

The terrestrial palynomorph record from Site U1356 represents the vegetation in the near-coastal lowlands and (to a lesser extent the hinterland) of the Wilkes Land sector of East Antarctica^40^. The mid-Miocene record is dominated by pollen of the southern beech (*Nothofagidites* spp.) and conifers (*Podocarpidites* spp.), both of which indicate woody subantarctic or sub-alpine vegetation (Fig. 2). Elements of shrub-tundra and peat-lands such as heather (*Ericipites* sp.), shrubs of the family Haloragaceae (*Haloragacidites* sp.), grasses (*Graminidites* spp.), Caryophyllaceae (*Colobanthus*-type), and *Sphagnum*-moss (*Stereisporites* sp.) are abundant, particularly after the MCO (Supplementary Data 5). High percentages of Podocarpaceae conifer pollen along with *Myricipites* (nearest living relative (NLR): Casuarinaceae) as well as regularly occurring tree ferns (NLR: *Cyathea*) suggest that even after the MCT a cold-temperate woody vegetation with shrubs and trees still existed in sheltered areas of the coastal lowlands. Temperature reconstructions derived from the fossil pollen assemblages suggest mean annual temperatures (MATs) between 5.8 and 13 °C, and summer temperatures >10 °C (Supplementary Table 2). At ~10.8 Ma, above the hiatus, a distinctively high abundances of *Nothofagus* and bryophytes accompanied by a general decrease in taxon diversity indicate cooling.

Comparison of Miocene pollen assemblages with Eocene and Oligocene species from the same Site U1356 reveals a different taxonomic composition in all samples. We therefore exclude the possibility of reworked Palaeogene pollen being re-deposited into these sediments^41^. We are also confident that most of the pollen, as well as any terrestrial organic matter (including soil), must have originated from close to the depositional site, as most pollen grains are well preserved and Antarctica was already isolated from surrounding landmasses at the Miocene time.

### Organic geochemical biomarkers

**Fig. 3:**
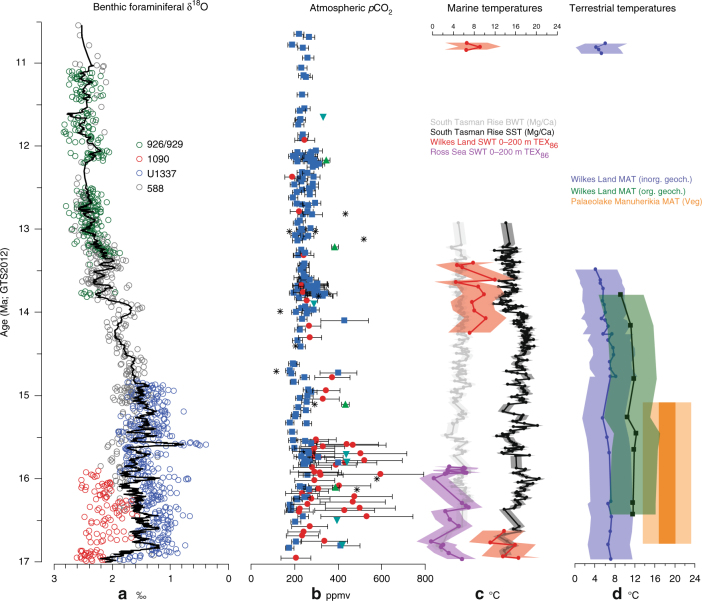
Southern high-latitude Miocene temperature and *p*CO_2_ change. **a** Deep-sea benthic foraminifer oxygen isotope record from ODP Sites 588, 926, 929, 1090 (see ref. ^12^), and IODP Site U1337^11^; **b** atmospheric *p*CO_2_ reconstructions^11–17^. Error bars represent uncertainties in underlying assumptions of each proxy; **c** bottom-water (BWT) and surface-water temperature (SST) reconstructions from Site 1171 South Tasman Rise^9^ (Mg/Ca, calibration error ±3 °C), seawater temperature (SWT) for Ross Sea AND-2A^21^ and Wilkes Land Site U1356 (${\mathrm{TEX}}_{86}^{\mathrm{L}}$, calibration error ±4 °C); **d** mean annual continental temperature (MAT) reconstructions for the Pacific sector of the Southern Ocean based on inorganic^53^ and organic geochemistry from Wilkes Land (calibration errors ±4 °C and ±5 °C, respectively) and macroflora remains (Veg.) from Palaeolake Manuherikia^28^ in New Zealand (calibration error ±5 °C)

Between 17 and 16.6 Ma, surface-water temperature reconstructions based on${\mathrm{TEX}}_{86}^{\mathrm{L}}$ (tetraether index of lipids consisting of 86 carbon atoms, polar calibration, 0–200 m water depth^42^) suggest temperatures of 11.2–16.7 °C (±2.8 °C calibration error) (Fig. 3, Supplementary Table 3, see Methods). The Branched vs. Isoprenoid Tetraether (BIT) index^43^ is widely used to estimate the soil organic matter input to the ocean. BIT indices of 0.4–0.8 are obtained for the samples between 16.4 and 14.8 Ma (Fig. 2, Supplementary Table 3). Values as high as 0.6–0.8 are at present found in coastal marine environments under substantial riverine influence^43^. While high BIT values prevented us from interpreting the${\mathrm{TEX}}_{86}^{\mathrm{L}}$ results^44^ during most of the MCO, they suggest high soil input and allow us calculating continental temperatures based on distribution of branched tetraether lipids^45^. Land temperatures are ~11.5 °C (±4.6 °C calibration error; Fig. 3, Supplementary Table 4).${\mathrm{TEX}}_{86}^{\mathrm{L}}$-derived temperatures are generally lower after 14.8 Ma, being on average 6 °C lower than during the MCO and highly variable, although some warm episodes are recorded. The BIT index is generally lower than 0.3 in this interval and continental temperatures derived from branched tetraether lipids could only be calculated for two samples at 14.2 Ma and 13.8 Ma, when temperatures are comparable (11 °C) or lower (9 °C), respectively, than those reconstructed for the interval >14.8 Ma.

## Discussion

The sedimentological, geochemical, and palynological results indicate that both the marine and terrestrial environments offshore Wilkes Land during the MCO (~17–14.8 Ma) were fundamentally different from that of today. Deposition occurred in an open-water environment where only occasional erosion or reworking of older marine sediments and transport to the core location took place. Open-marine surface waters were warm temperate, lacked a strong sea ice component, and were relatively low in nutrients compared to today. Specifically, dinocyst assemblages resemble those found today in the Pacific sector of the Southern Ocean at around or north of the Subtropical Fronts, where sea-surface temperatures vary between 8 °C and 17 °C^32^ (Fig. 1). The temperatures reconstructed with${\mathrm{TEX}}_{86}^{\mathrm{L}}$ (11.2–16.6 °C ± 2.8°C calibration error), albeit in a short interval between 17 and 16.6 Ma, fall within this range (Fig. 3).

**Fig. 4:**
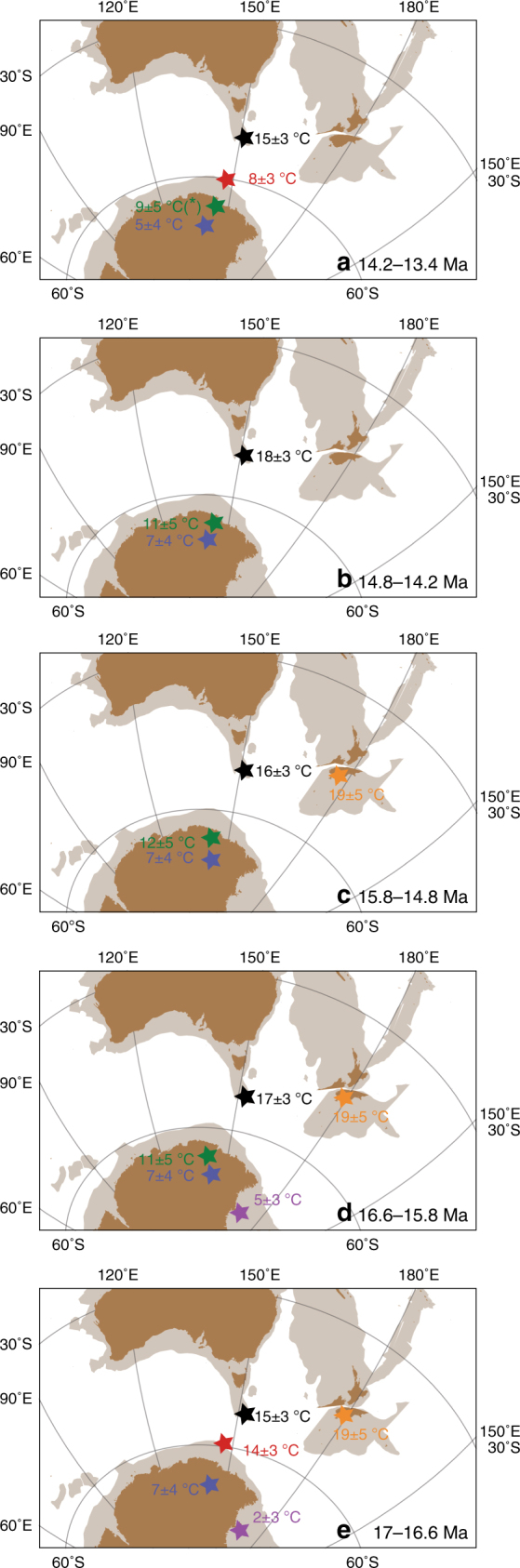
Southwest Pacific Latitudinal temperature gradient evolution during the mid-Miocene **a** 14.2–13.4 Ma; **b** 14.8–14.2 Ma; **c** 15.8–14.8 Ma; **d** 16.6–15.8 Ma; and** e** 17–16.6 Ma. Palaeogeographic reconstructions for 17 Ma (representative of the MCO, 17–14.8 Ma) and 13 Ma (representative of the interval 14.8–13.4 Ma) in a paleomagnetic reference frame^46^ derived from Gplates freeware^94^. Present-day landmasses are indicated in brown, continental lithosphere in light brown. Average ocean and continental temperatures (and relative calibration errors) calculated for different time slices. Black=Mg/Ca ocean surface temperature Site 1171; Red=TEX_86_^L^ ocean temperature Site U1356 based on organic geochemistry; Green=continental temperatures based on organic geochemistry Site U1356; Blue=continental temperatures based on inorganic geochemistry Site U1356^53^; Purple=TEX_86_^L^ ocean temperature AND-2A based on organic geochemistry^21^; Orange=continental temperature Palaeolake Manuherikia based on macroflora remains^28^ (* Temperature value is based on one sample only)

Site U1356 was located at about 59°S^46^ during the MCO, i.e., 4° more to the north than today (Fig. 4). This more northward position potentially facilitated the influence of warm, low-latitude-derived waters to Site U1356. Based on the present-day bedrock topography^47^ and reconstructions available for the Eocene–Oligocene^48^, Gasson et al.^22^ interpolated an approximate mid-Miocene Antarctic palaeotopography. This shows that a higher-than-present bedrock elevation of the now low-lying Wilkes Subglacial Basin would be able to accommodate a smaller Miocene marine-based ice sheet compared to today, and more bedrock exposure. Noteworthy, between 16.4 and 14.8 Ma, the high contribution of soil erosion material to Site U1356 (BIT index values up to 0.8, Fig. 2) implies that soil must have formed on extensive ice-free regions along the near-coastal lowlands of the margin and was eroded and transported to the drillsite. Possibly, the ice sheet was already dynamic and retreated to its terrestrial margin since (at least) the onset of the MCO, but additional warming from 16.4 Ma onwards would have enabled farther melting of continental ice and expansion of soil formation on the ice-free parts of the continent. Data on ocean temperature are missing, and ice extent is difficult to establish, but the ice-proximal AND-2A record shows no evidence of ice advance over the drillsite between 17 and 15.8 Ma and possibly until 14.6 Ma^20,21^. In the Wilkes Land record, three different proxies in the marine sediments indicate mild continental temperatures. Terrestrial palynomorphs suggests a temperate (MATs 5.8 and 13 °C, mean summer temperatures >10 °C), humid, locally ice-free coastal zone along the Wilkes Land margin covered by woody vegetation dominated by southern beech and Podocarpaceae conifers. Plant communities similar to that reconstructed from the Wilkes Land record have also been derived from Miocene pollen records near McMurdo Sound/Ross Sea^19,49^, McMurdo Dry Valleys^50^, and the Antarctic Peninsula^51^. However, in contrast to previously published records, the pollen assemblages here contain exceptionally high percentages of Podocarpaceae conifers and a greater diversity of woody taxa, suggesting warmer conditions at the Adélie Coast than at other Antarctic locations, with maximum temperatures around the MCO. Temperature based on branched tetraether lipids, likely reflecting mean annual temperature or mean temperature of growing degree days above freezing^52^, are 10–12 °C (±5 °C calibration error). An additional palaeotemperature proxy based on inorganic chemical weathering indices^53^ indicates comparable, albeit lower mean annual temperatures of 6–8 °C (±4 °C, Fig. 3).

Our data for the MCO interval suggest a seemingly persistent absence of a marine-based ice sheet at the Wilkes Land basin, a dynamic continental ice sheet profoundly retreated (on)to its terrestrial margin at peak warmth and ocean conditions warmer than those of today.

Our reconstructions for the post-MCO interval show a dynamic ice sheet and a fundamentally different environment compared to that of MCO. Wilkes Land Site U1356 (palaeolatitude ~60°S at 13 Ma^46^, Fig. 4) data indicate overall colder conditions than during the MCO, with high-nutrient surface waters, high productivity (dominance of protoperidinioid cysts), and sea ice presence, with common *Selenopemphix antarctica* (Fig. 2). Surface water temperatures are on average 6 °C lower than during the MCO, yet episodically reach peak values (Figs. 3 and 4). Noteworthy, coinciding with these peak temperature values, rapid increases in warm-water dinocyst taxa at 13.8 and 10.8 Ma with high percentages of *Nematosphaeropsis labyrinthus* (Fig. 2) suggest the presence of surface waters akin to those found today around the oceanic fronts north of the polar front^32^. The modern temperature preferences of *N. labyrinthus* 6–13 °C in winter and 8–17 °C in summer^32^ agree well with those reconstructed with${\mathrm{TEX}}_{86}^{\mathrm{L}}$. Interestingly, synchronous to the increases in *N. labyrinthus*, two pulses of outsized clasts (interpreted as ice-rafted debris (IRD)) at 14–13.8 and 10.8 Ma suggest that nearby glaciers were terminating into the marine system, much in contrast to the MCO for which such evidence is entirely lacking. Few icebergs reach the latitude of Site U1356 today, despite abundant marine-based ice sheets nearby due to deflection by the strong Polar Current. The episodic presence of extensive IRD at Site U1356 after the MCO thus requires the presence of marine-based ice sheets and an ocean circulation different from present. The location of Site U1356 at ~13 Ma was at 60°S, close to the present-day position of the Antarctic Divergence. If the ocean structure were similar to today, high surface water productivity would be expected, and heterotrophic protoperidinioid dinocysts would dominate the assemblages. However, episodic high abundance of gonyaulacoids temperature cysts and *N. labyrinthus*, which is at present associated to northern oceanic fronts, suggest a different oceanographic structure. A weakened circulation compared to that of present day is indicated allowing episodic southward transport of warm waters at Wilkes Land Site and icebergs to escape northward out of the Polar Current.

In this <14.8 Ma interval, both our marine palynological (via the high percentage of reworked dinocysts) and sedimentological proxy data indicate erosion of older sediments from the shelf, likely during ice advance and stronger current activity. A hiatus between 13.4 and 11 Ma and seismic profiles suggesting the migration of the channel levee systems depocentres in the region point to ice sheet expansion on the continental shelf^24^. The relative abundance of soil organic matter (BIT usually <0.3) in these sediments is generally low compared to those of the MCO, which is consistent with a colder, more productive ocean and less soil formation due to continental ice cover compared to the MCO. However, the pollen flora remains broadly unchanged, although higher abundance of the southern beech (*Nothofagus* spp.) and mosses (*Stereisporites/Coptospora*, Fig. 2) suggests lower continental temperatures than during the MCO, especially at 10.8 Ma. Geochemical proxies indicate MATs still reaching 6–8 °C (Fig. 3). Although relating cryosphere dynamics at the Wilkes Land subglacial basin with an offshore record inevitably requires assumptions that can only be tested through new drilling efforts even closer to the Antarctic continent, our data suggest that after the MCO, calving and retreat of the marine-grounded continental ice sheets occurred during episodic reoccurrence of warm waters close to the margin, while some refugia for vegetation persisted.

To further investigate the importance of the Southern Pacific Ocean surface oceanographic structure and temperature patterns in relation to the continental cryosphere dynamics during and after the MCO, we integrated our Wilkes Land record (MCO palaeolatitude ~59°S^46^) with existing Miocene reconstructions from the Ross Sea AND-2A record^21^ (MCO palaeolatitude: ~73°S^46^, Supplementary Table 5) and ODP Site 1171^9^ (MCO palaeolatitude: ~54°S^46^) south of Tasmania (Figs. 3 and 4). The AND-2A record indicates environmental conditions very different from today, particularly in two short exceptionally warm intervals at ~16.4 and ~15.9 Ma. The phototrophic temperate dinocyst *Operculodinium* cf. *centrocarpum* along with high freshwater algae and high pollen concentrations^19^, stable deuterium isotope data^54^, and surface seawater temperatures of ~7 °C^21^ (Fig. 3, Supplementary Table 6) are interpreted as episodes of reduced sea and continental ice^20^, increased hydrological cycling^54^, and mean air summer temperatures of at least 10 **°**C that sustained a tundra vegetation in the hinterlands^19^. In comparison to the Ross Sea, the Wilkes Land record provides evidence for a much warmer ocean throughout the MCO (Figs. 3 and 4) and absence of sea ice. Terrestrial palynomorphs also suggest continental conditions generally warmer than those reconstructed from the Ross Sea. Such a temperature gradient between the Wilkes Land and the Ross Sea records is well compatible with the fact that the Ross Sea is located further south in an ice-proximal location compared to Wilkes Land ice distal conditions already during the Miocene^46^. Possible mechanisms facilitating the occurrence of warm surface waters close to the Antarctic continent are the upwelling of (proto-) Circumpolar Deep Waters^55^ (CDW), if circulation was similar to that of today, or south displacement of warm surface waters. A weak (proto-) Antarctic polar front could possibly explain the movement of warmer waters from the north. The Southern Ocean circulation, the Antarctic Circumpolar Current (ACC), the front developments, and the upwelling of warm CDW depend on the wind patterns and topography, conditions for which there are poor constraints for the Miocene.

A comparison between the Wilkes Land and the Southern Tasmanian ODP Site 1171 records shows comparable surface seawater temperatures (within the calibration errors of both methods) during the MCO (Fig. 3) that are several degrees higher than those in the Ross Sea. Even when considering the closer distance between the Tasmanian and Wilkes Land sites compared to today (5° during the MCO^46^ as opposed to 15° today), the latitudinal temperature gradient between both sites appears strongly reduced, with the Wilkes Land site recording exceptionally warm surface seawater. A much-reduced temperature gradient between the two sites could exist if the ACC and its oceanic fronts were weaker compared to today and/or the ACC was displaced southwards.

The exact timing of a modern-strength fully developed ACC is still uncertain, but usually indicated between 41 and <23 Ma^56–59^, although younger ages have been proposed^60^. As the modern Southern Ocean circulation was possibly developed during the MCO, oceanic fronts were in place. The ACC and associated fronts are very sensitive to seafloor topography^61^, and a different topography may have had consequences for latitudinal heat transport. During the MCO the Tasmanian Gateway was narrower than at present (Fig. 4). A shift of the subtropical front equatorwards and subantarctic front poleward was reconstructed for the warm Pliocene of the Southern Ocean^62^. A comparable shift during the MCO would indeed make ODP Site 1171 and the Wilkes Land site been bathed by water masses with similar temperatures. A reduced surface-temperature gradient between offshore Adélie Coast and the South Tasman Rise could have been caused by weakened oceanographic fronts. Our data document the absence of substantial sea ice at the Wilkes Land site during the MCO, which, together with melting of the continental cryosphere, may have created surface water stratification. Hence, bottom-water formation at that time must have been limited to the coldest parts of the Antarctic shelves (e.g., the Ross Sea). In conclusion, our data infer a fundamentally different oceanographic and environmental setting across the Pacific sector of the Southern Ocean during the MCO compared to the modern day.

As Southern Ocean circulation is driven by winds and hence highly depends on patterns of atmospheric circulation, we compared the available information on continental climate and temperature gradients across the Pacific sector of the Southern Ocean. Continental temperatures reconstructed using leaf physiognomy at Palaeolake Manuherikia (mid-latitude New Zealand^28^) show mean annual values of 16.5–20 °C (Figs. 3 and 4), with amplified seasonal contrast in both temperature and precipitation during the MCO compared to present day. Such a seasonal gradient can be achieved in an ocean-moderated climate and can be caused by shifts in the position of the subtropical pressure cells with a westerly wind belt receding to the south compared to today during the summers. Continental mean annual temperatures obtained at Wilkes Land of ~10 °C and summer temperature estimates of 10 °C for the Ross Sea indicate that a clear latitudinal continental temperature gradient existed between the Antarctic continent and mid-latitude New Zealand, but yet much reduced compared to today.

After the MCO, maximum ice sheet extension is recorded at the AND-2A location in the Ross Sea^21^. The latitudinal ocean temperature gradient between the Wilkes Land and Site 1171 increased compared to MCO, but never reached that of present day. The cooling offshore Adélie Coast and the formation of sea ice after the MCO is broadly synchronous with decreasing atmospheric CO_2_ concentrations (Fig. 3). However, our reconstructions support a scenario with cold-temperate conditions marked by a periodic reoccurrence of warmer, sea ice-free conditions. Our data indicate a substantial increase in ice sheet discharge during contact with warmer waters, most likely associated with a collapse of the marine-based ice sheet, likely sensitive to ocean temperatures.

In conclusion, profound ice sheet retreat at the Wilkes Land subglacial basin further than its land-terminating margin occurred during the MCO, in association with a warm surface ocean, an oceanographic regime fundamentally different from that of today and with CO_2_ concentrations generally similar to those expected for the near future in a “business as usual scenario”^63^. After the MCO, the Wilkes Land margin cooled, sea ice formed, but ocean waters and continental conditions remained relatively mild. The continental cryosphere, which extended into a marine-based ice sheet, was still substantially reduced compared to the present day even at pre-industrial atmospheric CO_2_ values. Episodic reoccurrence of warm waters, within an oceanographic configuration still different from that of present day, destabilised the marine-based ice sheet, leading to melting and iceberg discharge. This demonstrates a strong coupling of oceanic climate and Antarctic continental conditions, and highlights the important role of the ocean for the stability of the cryosphere.

Our results further confirm and may be taken to extrapolate recent monitoring observations of the East Antarctic ice sheet, which indicate the high sensitivity of the Wilkes subglacial basin to ocean warming.

## Methods

### Particle size/IRD

Particle-size analyses were performed at the Department of Earth and Environmental Studies, Montclair State University. Samples were prepared using standard operation procedures outlined in ref. ^64^. Samples were mechanically and chemically disaggregated through ultrasonic treatment and heating with 30% hydrogen peroxide and 10% HCl. Samples were dispersed through addition of sodium pyrophosphate and the solutions were heated to allow all dispersant to dissolve. A Malvern Mastersizer 2000 laser particle sizer was used to measure the grain-size distributions of the samples. Instrument settings were based on the recommendations of ref. ^65^. Industrial and natural standards were monitored for quality control. IRD counts were carried out shipboard; counts were normalised per metre of recovered section length at the Department of Earth and Environmental Studies, Montclair State University.

### Age model

We developed the age model to U1356, using an integrated biostratigraphic and magnetostratigraphic methodology similar to that presented in Tauxe et al.^29^. Radiolarian and diatom first and last appearance datums (FADs and LADs) were derived using Constrained Optimisation methodology as presented in Crampton et al.^30^ for diatom turnover events in the Southern Ocean. We apply the same methodology but use both radiolarian and diatom data. CONOP generates a parsimonious, best-fit composite sequence of biostratigraphic events of radiolarian and diatom derived from 36 cores in the Southern Ocean, to which observed biostratigraphic events in U1356 could be placed. This paper uses the “hybrid range” model, which was shown by Cody et al.^66^ to be most suitable for sites close to the Antarctic continental margin. We use two versions of this model, a “strict” and “relaxed” version, which prohibits or allows for FAD and LAD contraction to move out of its observed range as a correlation is made between U1356 observations and the composite sequence. Full details on this methodology are discussed in Cody et al.^66^ and Crampton et al.^30^. To represent uncertainties, we apply the spread of the ages from all CONOP-placed FAD and LAD data in these two models at every depth where there is an observed biostratigraphic event. This is in contrast to the approach used by Tauxe et al.^29^, which assigned absolute values for FADs and LADs as were presented in the CONOP Average age range model of Cody et al.^67^. However, Cody et al.^66^ showed that the Hybrid age model converged upon a robust age model earlier than the Average age range model and thus is more appropriate to use. The magnetostratigraphy presented in Tauxe et al.^29^ is revised on the basis of these new CONOP constraints. We also include palynology-based FADs presented in Tauxe et al.^29^, and note there is now an improved fit between the various chronological datasets.

### Palynology

Sample processing was performed at Utrecht University, following standard techniques of the Laboratory of Palaeobotany and Palynology. Samples were oven-dried and weighed (~15 g dry weight sediment each), and one *Lycopodium clavatum* tablet with a known amount of marker spores (Batch #: 483216; 18,583 ± 4.1% spores per tablet) was added for quantification of palynomorph abundances^68^.

Samples were treated with 10% HCl (Hydrochloric acid) and cold 38% HF (Hydrofluoric acid), and sieved over a 10 µm mesh with occasional mild ultrasonic treatment. To avoid any potential processing-related preservation bias, no oxidation was carried out. The processed residue was transferred to microscope slides using glycerine jelly as a mounting medium, and 2–3 slides were analysed per sample at 400× magnification. Slides were used for both marine (dinocysts, acritarchs, other aquatic palynomorphs) and terrestrial (pollen and spores) palynological analyses. Dinocysts were identified based on a taxonomical index^69^ and informally and formally described species in the literature^36–38,70^. Of the 31 palynological samples analysed for dinocysts, 11 were either totally or almost barren (yielding only 15–25 dinocysts). These samples are still considered in our dataset; however, because of the low dinocyst yield, careful interpretation is required for these samples. Dinocyst percentages were calculated based on the total in situ dinocysts counted, excluding reworked specimens (Supplementary Data 4). Protoperidinioid (P) dinocysts are mostly represented by the genera *Brigantedinium*, *Lejeunecysta*, and *Selenopemphix*. Gonyaulacoid (G) dinocysts mostly include *Impagidinium* spp., *Operculodinium* spp., *Batiacasphaera* spp., *Nematosphaeropsis labyrinthus*, and *Spiniferites/Achomosphaera* spp. Protoperidinioid cyst percentages were calculated to identify productivity trends, as P dinocysts are likely produced by heterotrophic dinoflagellates^71^, while G are generally produced by phototrophic dinoflagellates. Reworked dinocysts include Eocene and Oligocene taxa (such as *Deflandrea* spp., *Enneadocysta diktyostila*, and *Vozzhennikovia* spp.). Reworked dinocyst percentages, calculated based on a sum comprising both in situ and reworked dinocysts, vary between 0% and 34%. In situ dinocyst absolute abundance (dinocysts/g dry weight, Supplementary Data 4) was calculated by counting the amount of *Lycopodium clavatum* spores encountered and following the equation of Benninghoff^72^.

Of the 31 samples analysed for pollen and spores, 15 were productive, and total counts range between 80 and 210 pollen and spores (Supplementary Data 5). Also, 33 in situ and 12 reworked pollen and spore taxa were identified from the literature^49,73^. Percentages of reworked pollen and spores were calculated based on the sum of total pollen and spores. For calculation of in situ palynomorph percentages, reworked pollen and spore counts were excluded from the total sum. Reworked pollen and spores have been identified using visual colour and fluorescence microscopy^41^. Permian to Palaeogene reworked pollen and spores have a dark, yellowish colour indicating geothermal maturity, and were clearly distinguishable from well-preserved in situ palynomorphs.

We reconstructed terrestrial MAT and mean summer temperature using the coexistence approach (CA)^74^ (Supplementary Table 2). The CA uses the climatic requirements of the NLR of fossil taxa to reconstruct the past climatic range, and is based on the assumption that the climatic requirements of the fossil taxa are similar to those of their NLRs. The CA produces a temperature range that comprises the climate interval in which all taxa of the reconstructed palaeo-vegetation can co-exist.

### Present-day dinoflagellate cyst assemblages and ecology

Present-day ecological preference of dinocysts is based on the analyses of more than 2400 globally distributed surface sediment samples^33^. Prebble et al.^32^ augmented the information available from the Southern Ocean by increasing the number of samples analysed to 311. We use their data to derive present-day dinocyst assemblages across the Southwest Pacific fronts and produced the data presented in Fig. 1. Prebble et al.^32^ analysed the relationships between assemblages and sea-surface temperature, water column depth, and productivity by means of ordination techniques and identified seven clusters. One of these clusters (Cluster 7) groups the South Atlantic samples only, north of the STF and is not considered here. Data from the other six clusters are presented in Supplementary 1. All the species identified in the surface samples have been placed in this study into 6 groups: *Selenopemphix antarctica* (SA), other protoperidinioids (oP), *Impagidinium* spp. (I), *Nematosphaeropsis labyrinthus* (Nl), *Operculodinium* spp. (O), and other Gonyaulacoids (oG) and are presented in percent relative to the total assemblage. These six groups are chosen either based on specific ecological preferences or because of their cosmopolitan occurrence^33^. Importantly, dinocysts in our Miocene record can also be categorised in the same six groups. In this way, we aimed to minimise the uncertainties in the environmental reconstructions potentially caused by extinct Miocene species, although we cannot discard the possibility that some taxa may have partly changed their preferred ecological niche through time^35^. However, we defer from quantitatively interpreting the changes observed and use the modern dinocyst distribution only as a qualitative tool. The percentages presented in Fig. 1 represent the median percentage of the taxa found (Supplementary Table 1). In this modern dataset, sea-surface temperature accounts for 38–56% of the variation. However, modern assemblages are also sensitive to other water-column parameters such as productivity and distance from shore. Notably, Cluster #2 contains almost exclusively protoperidinioid cysts; and all samples in this cluster come from South Atlantic and East Pacific locations around the STF and SAF and slightly south of the Chilean upwelling, which are all areas of high productivity. Only three samples from Cluster #2 are from within the STF region east of New Zealand.

### Organic geochemical biomarkers

Twenty-nine powdered and freeze-dried sediments (~15 g dry weight) were extracted with dichloromethane (DCM)/methanol (9:1) using the Dionex accelerated solvent extraction (ASE) technique. The extracts were separated by Al_2_O_3_ column chromatography using hexane/DCM (9:1), 100% DCM, and DCM/methanol (1:1) to yield the apolar, ketone, and polar fractions, respectively. The ketone fractions were checked for alkenones, but none of the samples were found to contain them. The polar fractions were analysed for tetraether lipids and were used to calculate the ${\mathrm{TEX}}_{86}^{\mathrm{L}}$^42,75^ the branched vs. isoprenoid tetraether BIT^43^, and the relative distribution of branched tetraether lipids used to estimate mean annual temperature^45^. The TEX_86_ ratio is based on isoprenoidal–glycerol dialkyl glycerol tetraether (GDGTs)^76^, which are assumed to derive mainly from Thaumarchaeota, formerly known as Crenarchaeota group 1^77^, which is an abundant group of marine Archaea. The polar fractions were dissolved in a 99:1 hexane/propanol solvent, and were filtered using a 0.45 μm, 4 mm diameter polytetrafluoroethylene filter, before being analysed using a high-performance liquid chromatography/atmospheric pressure positive ion chemical ionisation mass spectrometry (HPLC/APCI-MS) as described by Schouten et al.^78^. Extractions were performed at Utrecht University, and HPLC/MS analyses were carried out at the NIOZ (Royal Netherlands Institute for Sea Research).

For conversion to temperature we use following equation:

1 $${\mathrm{Sea}}\,{\mathrm{Water}}\,{\mathrm{Temperature}}\,\left( {{\mathrm{SWT}}} \right) = {50.8} \ast {\mathrm{TEX}}_{86}^{\mathrm{L}} + {36.1}\left( {r}^{2} = {0.87};\,{n} = {396} \right)$$

which is best suited for polar oceans, and used the calibration of Kim et al.^42^ for water depth of 0–200 m. The analytical error is 0.3 °C, while the uncertainty introduced by the calibration error for ${\mathrm{TEX}}_{86}^{\mathrm{L}}$ in the subsurface (0–200 m) calibration is estimated at ±2.8 °C^42^ (error propagation used in Fig. 4 is ±3 °C). This calibration was chosen for three main reasons: Thaumarchaeota in Antarctic waters are especially abundant in the winter cold and salty waters at depths of ~ 45–100 m^79^; Application of the 0–200 m calibration to a Holocene sediment record close to the studied Wilkes Land location revealed known climate variations and reasonable absolute temperature estimates^42^; Seawater temperatures are compared in this study with those obtained from Mg/Ca analyses of the foraminifer *Globigerina bulloides* from ODP Site 1171 in the Southwest Pacific offshore Tasmania. Analytical error for Mg/Ca in *Globigerina bulloides* is ±~ 1 °C, while the error, which considers other processes such as uptake of Mg/Ca and composition of seawater, is ~±3 °C^9^ (error propagation in Fig. 4 is ±3 °C). Recent core-top sediments studies carried out in the Southwest Pacific Ocean (33–54°S) have shown that temperature reconstructions obtained with Mg/Ca on *G. bulloides* correlates best with water temperatures at 200 m depth^80^.

Although the seasonal abundance of Thaumarchaeota may be higher in winter, ${\mathrm{TEX}}_{86}^{\mathrm{L}}$ temperature reconstruction may possibly still be skewed towards the summer season particularly in polar areas because of the more efficient food web-based scavenging of thaumarchaeotal cells due to the higher summer productivity.

For comparison SST (0 m depth) values based ${\mathrm{TEX}}_{86}^{\mathrm{L}}$ are also reported following the equation of ref. ^75^

2 $${\mathrm{SST}} = {67.5} \ast {\mathrm{TEX}}_{86}^{\mathrm{L}} + {46.9.6}\left( {{r}^{2} = {0.87};\,{n} = {255}} \right),$$

The uncertainty introduced by the calibration error is in this case ±4 °C. We calculated the SST based on the ${\mathrm{TEX}}_{86}^{\mathrm{H}}$ 0–200 m calibration of Kim et al.^42^ and for the Bayesian calibration of TEX_86_^81^. These two calibrations produce similar values compared to each other, but are up to 8.5 °C degrees higher than the ${\mathrm{TEX}}_{86}^{\mathrm{L}}$ calibrations, although the down-core trend remains approximately the same (data not shown). ${\mathrm{TEX}}_{86}^{\mathrm{L}}$ has a strong depth dependence^82^ due to changes in the GDGT 2/3 ratio. However, similar trends between ${\mathrm{TEX}}_{86}^{\mathrm{H}}$ and ${\mathrm{TEX}}_{86}^{\mathrm{L}}$ suggest that this has not been a major issue in our Miocene sediment record. In view of these uncertainties, absolute values obtained with ${\mathrm{TEX}}_{86}^{\mathrm{L}}$ should be interpreted with care and in concert with other data obtained from dinoflagellate cysts.

BIT index^43^ is used as indicator for continental organic matter input. A large input of organic matter from the continent also carries isoprenoid GDGTs, which can mask the autochthonous TEX_86_ signal^83^. We discarded ${\mathrm{TEX}}_{86}^{\mathrm{L}}$-temperature estimates in our samples with BIT >0.3^44^.

Consequently, 10 of the 29 samples measured in the Wilkes Land record were excluded from the ${\mathrm{TEX}}_{86}^{\mathrm{L}}$ data points (Supplementary Table 3), and only the remaining temperature values are plotted in Figs. 2 and 3. BIT index values in the AND-2A record (Supplementary Table 6) are mostly <0.3.

TEX_86_ can be further biased by an input of GDGTs derived from methane-utilising Archaea^84,85^. Furthermore, methane-generating Archaea, which mainly produce GDGT-0 but also minor amounts of GDGTs 1–3, can alter the TEX_86_ signal. The “Methane Index” can be calculated^86^, which evaluates the contribution of methanotrophic archaea to the total GDGT pool:

3 $$\begin{array}{*{20}{l}} {{\mathrm{MI}}} \hfill &  = \hfill & {[{\mathrm{GDGT}} - 1] + [{\mathrm{GDGT}} - 2]} \hfill \\ {} \hfill & {} \hfill & { + [{\mathrm{GDGT}} - 3]/\left[ {{\mathrm{GDGT}} - 1} \right]} \hfill \\ {} \hfill & {} \hfill & { + [{\mathrm{GDGT}} - 2] + [{\mathrm{GDGT}} - 3] + \left[ {{\mathrm{Crenarchaeol}}} \right] + [{\mathrm{Cren}}\,{\mathrm{isomer}}]} \hfill \end{array}.$$

Values of 0.3–0.5 of MI mark the boundary between “normal marine sediments” and methane-impacted sediments. Calculations of this MI index for the studied Wilkes Land record vary between 0.05 and 0.23, in the samples for which temperatures are calculated and used, and between 0.03 and 0.06 for the AND-2A record (Supplementary Table 3 and 6, respectively). The values indicate that there is no temperature bias due to an input of methanogenic or methanotrophic archaea.

Finally, we calculated the Ring Index^87^

4 $$\begin{array}{ccccc}\\ {\mathrm{Ring}}\,{\mathrm{Index}} = & 0 \ast \left[ {{\mathrm{GDGT}} - 0} \right] + 1 \ast \left[ {{\mathrm{GDGT}} - 1} \right] + 2 \ast \left[ {{\mathrm{GDGT}} - 2} \right]\\ \\ & + 3 \ast \left[ {{\mathrm{GDGT}} - 3} \right] + 4 \ast \left[ {{\mathrm{Crenarchaeol}}} \right]\\ \end{array}.$$

We compared this with the theoretical prediction of the Ring Index based on the global correlation of this index with the TEX_86_ and found that the remaining ${\mathrm{TEX}}_{86}^{\mathrm{L}}$ values used in this study were all within 0.6 (ΔRI) and thus fitting the observed trend of increasing Ring Index with TEX_86_.

Based on the distribution of branched GDGTs (brGDGTs), annual MAT can be estimated in samples where high BIT index values (>0.3). For this, the calibration of Peterse et al.^88^ can be used (Supplementary Table 4):

5 $${\mathrm{MAT}} = 0.81 - 5.67 \ast {\mathrm{CBT}} + 31.0 \ast {\mathrm{MBT}}^\prime \left( {r^2 = 0.59,\,n = 176} \right)$$

where MBT’ is the methylation index based on the seven most abundant GDGTs and CBT. The uncertainty introduced by the calibration error is estimated to be ±5.0 °C.

However, newer methods and calibrations have recently become available^45,52^. For this, samples with high BIT indices were rerun using improved chromatography of brGDGTs^89^. Temperature were estimated using the calibration of De Jonge et al.^45^:

6 $$\begin{array}{ccccc}\\ {\mathrm{MATmrs}} 	 = 7.17 + 17.1 \ast \left[ {Ia} \right] + 25.9 \ast \left[ {Ib} \right]\\ \\ 	 + 34.4 \ast \left[ {Ic} \right] - 28.6 \ast \left[ {IIa} \right]\left( {r^2 = 0.68,\,n = 222} \right)\\ \end{array}.$$

The uncertainty introduced by the calibration error is ±4.6 °C (indicated as ±5.0 °C in Fig. 4).

Of the 10 samples rerun, 2 had brGDGT concentrations below detection limit and were discarded. Finally, we also report values from the newest calibration by Naafs et al.^52^ (Supplementary Table 4). These MATs show the same trend but a much higher variability due to the high variability in 5 methyl brGDGTs vs. 6 methyl brGDGTs in our samples. Since this large temperature variability contrasts with reconstructions obtained from vegetation, we here used temperature obtained with the calibration of De Jonge et al.^45^.

### Data availability

All data generated for this study are included in this article (and its Supplementary Information files). Original raw data (palynology counts and (br)GDGTs concentrations and chromatograms) are available from the corresponding author upon request.

## Electronic supplementary material

Supplementary Information

Description of Additional Supplementary Files

Supplementary Data 1

Supplementary Data 2

Supplementary Data 3

Supplementary Data 4

Supplementary Data 5
