# Supplementary material for: Southern Ocean warming and Wilkes Land ice sheet retreat during the mid-Miocene
Source: Nat Commun. 2018 Jan 22;9:317. doi: 10.1038/s41467-017-02609-7 (PMC5778126; doi:10.1038/s41467-017-02609-7)
Supplement: Supplementary file 1 — Supplementary Information [file 41467_2017_2609_MOESM1_ESM.pdf]

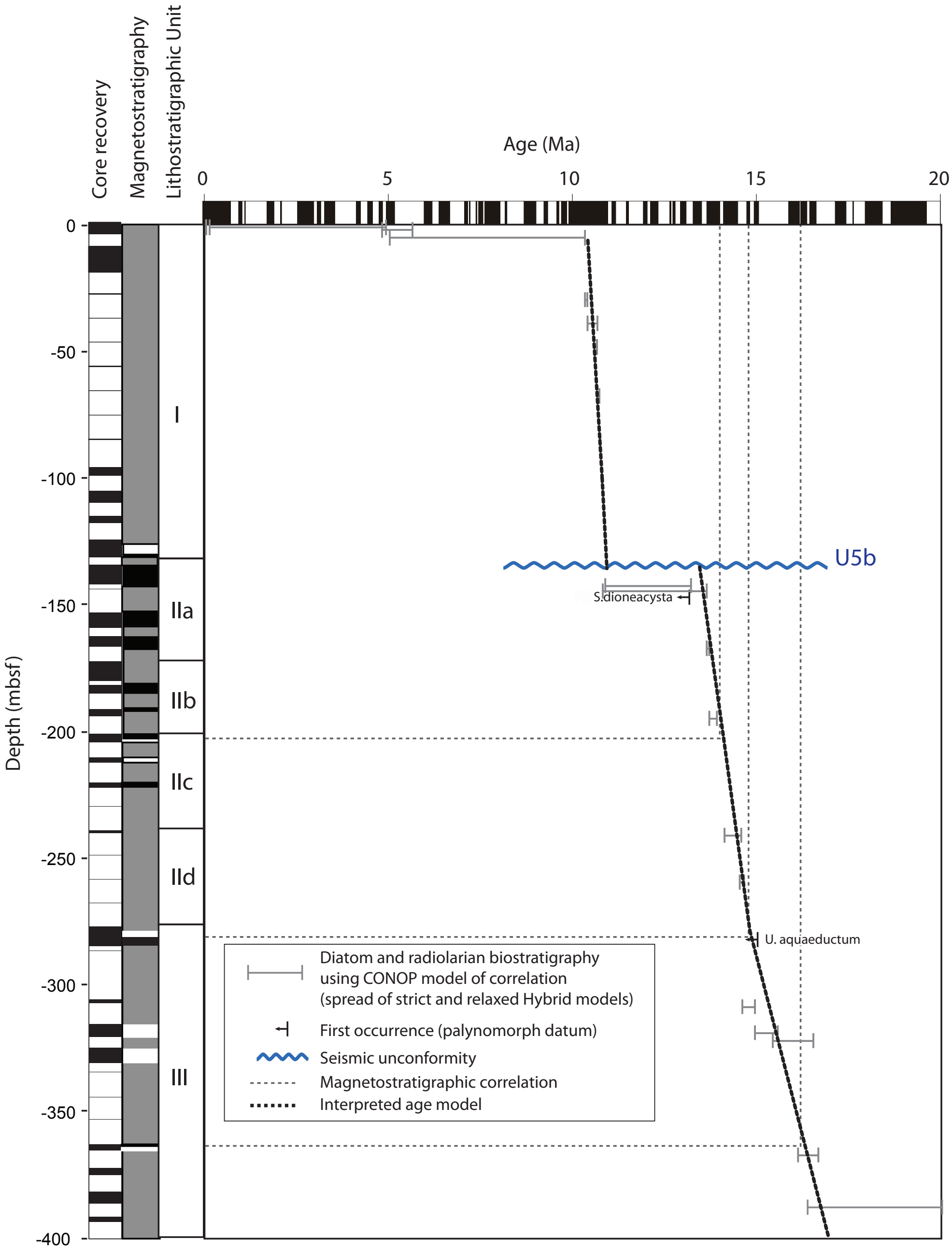

Supplementary Figure 1: Age model for the Miocene sediments of IODP Hole U1356A. Core recovery and lithostratigraphic units are shown. Magnetostratigraphy and palynology tie points are taken from Tauxe et al.[1] Sediments are covered above a hiatus that spans most of the early Miocene[1]. Diatom and radiolarian biostratigraphic tie points are derived from the CONOP model of correlation[2,3] (Supplementary Data 1 and 2). All ages are given following the GTS2012 time scale[4].

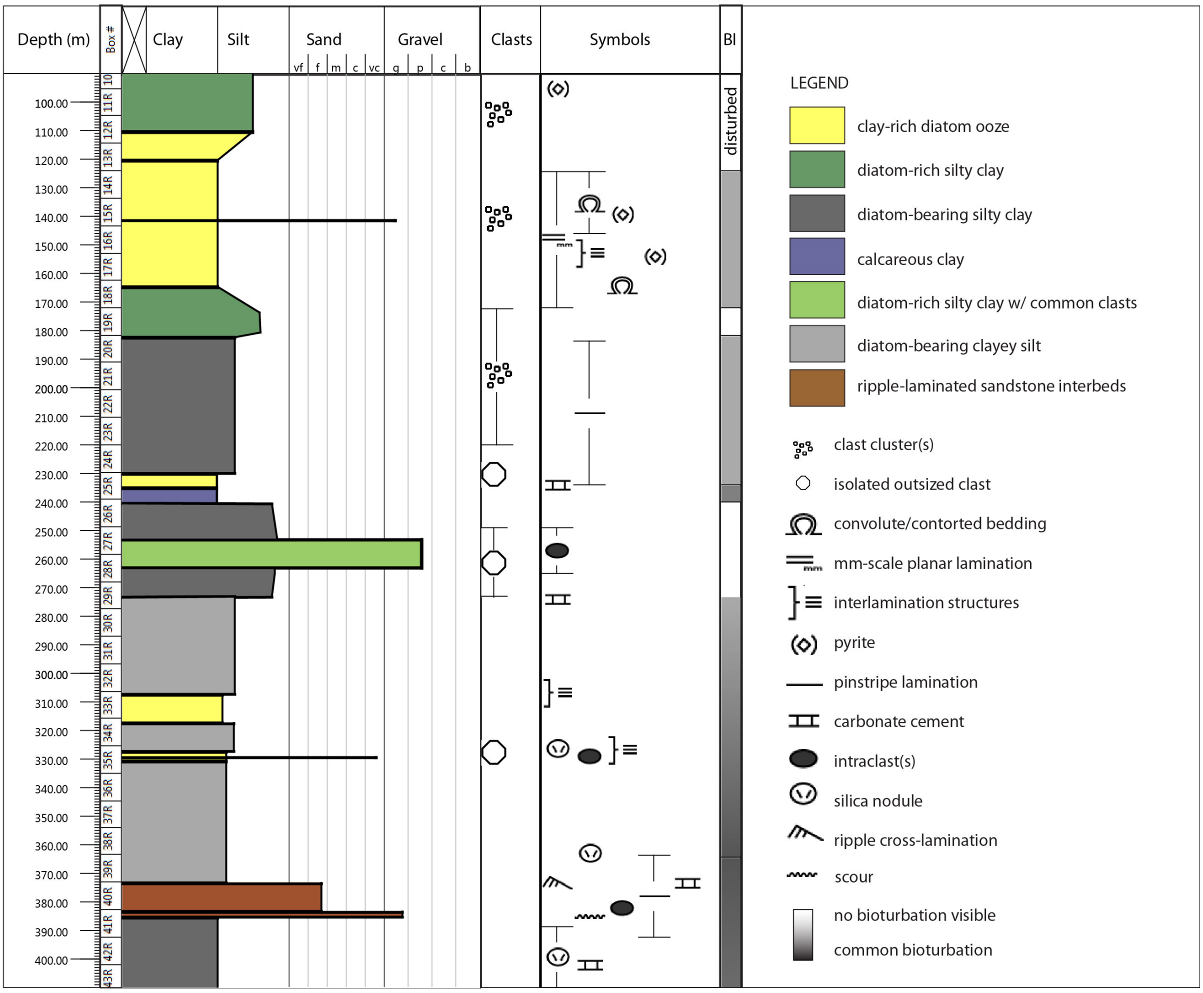

Supplementary Figure 2: Lithostratigraphic log (updated from the shipboard log5) for the Miocene portion of Site U1356.

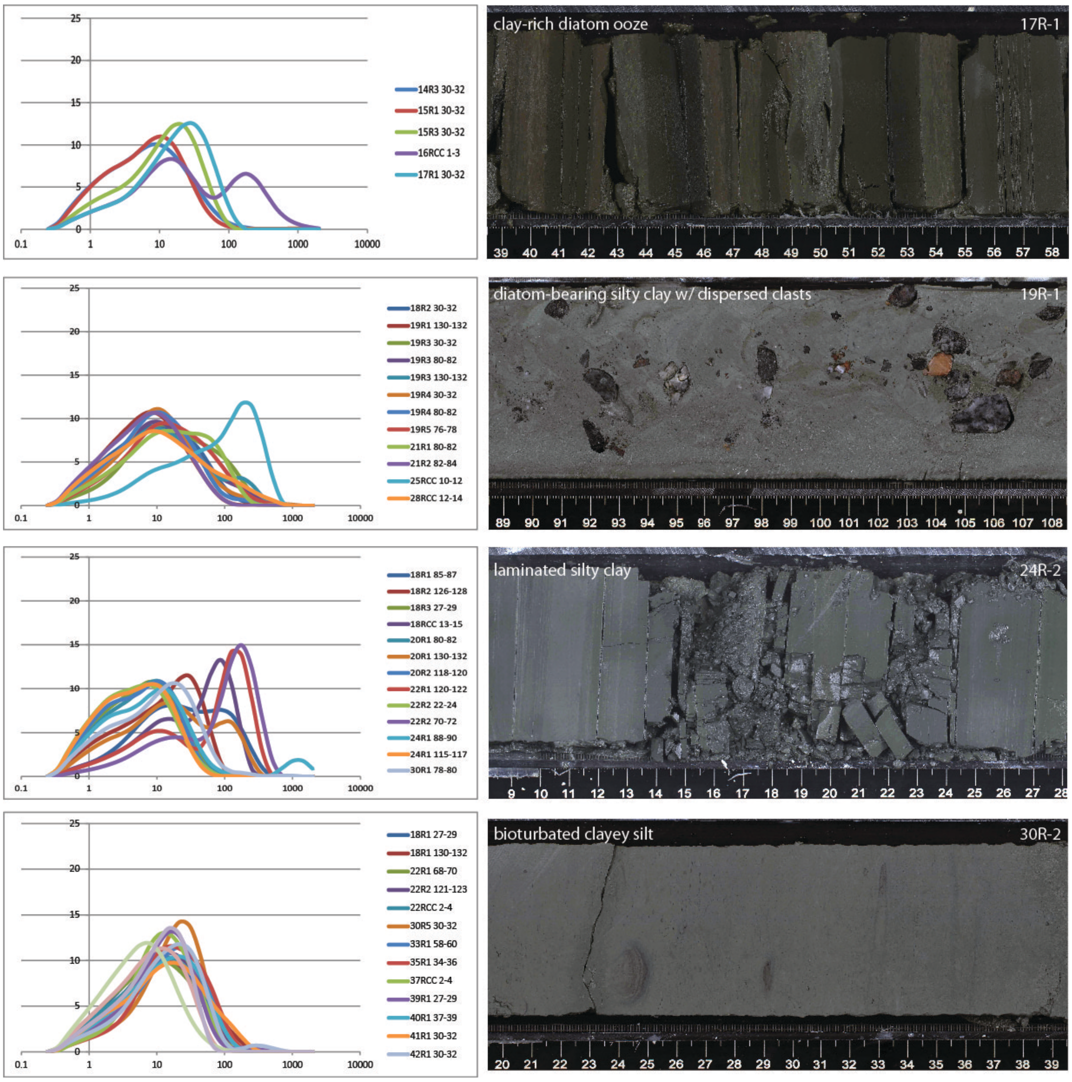

Supplementary Figure 3: Representative facies and grain-size distributions for the Miocene section of Hole U1356A. The digital color images were obtained on board the Joides Resolution immediately after the cores were split with a line-scan camera (AF Micro Nikon lens; 60mm; 1:2:8 D) mounted on a track. We acknowledge IODP, the JOIDES Resolution Science Operator (JRSO), and the Shipboard Scientific Party. Images can be downloaded at http://publications.iodp.org/proceedings/318/EXP_REPT/CORES/IMAGES/

Cluster # (n)	Tmed	Tmin	Tmax	Pmed	Pmin	Pmax	Sa	oP	I	Nl	O	oG
1 (11)	0.7	-1.3	3.6	502.3	127	928	90	8.2	1.8	0	0	0
2 (50)	9.5	0.4	20.4	338	0	1275	0	99.2	0	0.8	0	0
3 (45)	12.7	7.3	27	836.6	98.5	1810	0	16	8.5	59.3	10.7	5.5
4 (92)	15.1	1.8	27.2	571.5	68.4	3367	0	81.9	0	6.7	9.7	1.7
5 (50)	17.1	10.4	20.4	707.4	100.2	2005	0	2.5	76.2	15.3	2.9	31.1
6 (16)	18.5	14.0	24.2	539	152.7	3816	0	4.1	0	0	90.1	5.8

Supplementary Table 1: Percentages of the most representative dinocyst taxa or groups found in present-day sediment samples from the Southern Ocean[6] see also Methods).
Cluster number (#) with the total amount of samples analysed per cluster (in brackets). Median, minimum and maximum temperature (T) values in °C and median, minimum and maximum productivity (P) in gCm^2d^{-1}. Dinocysts: Sa = *Selenopemphix antarctica*; oP = other Protoperidinioids; I = *Impagidinium* spp.; Nl = *Nematosphaeropsis labyrinthus*; O = *Operculodinium* spp.; oG = other Gonyaulacoids. Pie charts representing dinocyst percentages in surface samples assemblages are presented in Figure 1.

Fossil Taxon	Nearest Living Relative	Ref. for NLR	NLR used for climate analysis	MAT-min	MAT-max
Baculatisporites comaumensis	Osmundaceae, Hymenophyllaceae	7	Osmundaceae, Hymenophyllaceae	3.7	27.3
Caryophyllidites sp.	Caryophyllaceae	7	Caryophyllaceae	3.4	29.6
Chenopodipollis chenopodiacides	Chenopodiaceae	7	Chenopodiaceae	5.1	29.6
Coptospora	Bartramiaceae, *Conostomum*	8	*Conostomum*	3.4	13.7
Cyathidites	Cyatheaceae (Cyathea), Dicksoniaceae, Schizaeaceae (Lygodium);	7	Cyatheaceae	5.8	27.3
Ericipites longisulcatus	Ericaceae	7	Ericaceae	3.4	29.2
Gleicheniidites senonicus	Gleicheniaceae	7	Gleichenia	4.9	28.8
Haloragacidites	Haloragaceae	7	Haloragaceae	3.4	29.4
Liliacidites intermedius	Liliaceae	7	Liliaceae	4.5	27.5
Lymingtonia cenozoica	Nyctaginaceae, Portulacaceae cf. *Montia*	7	*Montia*	3.4	22.9
Microcachryidites antarcticus	*Microcachrys tetragona, Microstrobos*	7	*Microcachrys tetragona*	4.4	13.7
Myricipites harrisii	Casuarinaceae, possibly also Myricaceae	7	Casuarinaceae (all Australian species)	5.1	28
Nothofagidites flemingii	Nothofagaceae (*Nothofagus*)	9	Nothofagaceae	4.7	24.1
Nothofagidites lachlaniae	Nothofagaceae (subg. *Fuscospora)*	9	Nothofagaceae	4.7	24.1
Nothofagidites sp.	Nothofagaceae *(Nothofagus)*	9	Nothofagaceae	4.7	24.1
Nothofagus brassii	Nothofagaceae *(Nothofagus)*	9	Nothofagaceae	4.7	24.1
Peninsulapollis gilii	Proteaceae	7	Proteaceae	3.4	29.6
Phyllocladidites mawsonii	*Lagarostrobos*	7	*Lagarostrobos*	5.7	13.7
Podocarpidites sp.	Podocarpaceae *(Podocarpus)*	7	*Podocarpus*	3.4	27.4
Proteacidites sp.	Proteaceae	7	Proteaceae	3.4	29.6
Ranunculaceae	Ranunculaceae	7	Ranunculaceae	3.4	28.1
Rudolphisporis rudolphi	Anthocerotaceae (cf. *Anthoceros)*	7	Anthocerotaceae	6.2	27.5
Stereisporites sp.	*Sphagnum*	10	*Sphagnum*	3.4	27
Tricolpites reticulatus	Gunneraceae *(Gunnera)*	7	*Gunnera*	4.8	13.8
Tubulifloridites sp.	Asteraceae subf. Tubuliflorae	7	Asteraceae	3.4	29.6

Supplementary Table 2 - Nearest Living Relative (NLR) and MAT range for each fossil taxon used for the Coexistance Approach (see Methods).

IODP Hole	Core	Sector	Depth (cm)	Depth (mbsf)	Age (Ma)	TEX_{86}	SST (0 m)	SWT (0-200m)	BIT index	MI	GDGT -0/cren	Ring Index	ΔR
U1356A	11R	2W	20-22	97.1	10.77	0.43	7.8	6.7	0.1	0.07	1.09	1.91	-0.03
U1356A	12R	3W	20-22	108.1	10.81	0.51	11.3	9.3	0.1	0.05	1.13	1.88	0.17
U1356A	13R	2W	20-22	116.2	10.85	0.42	7.8	6.7	0.1	0.09	1.16	1.84	0.01
U1356A	15R	1W	20-22	134.0	13.41	0.52	9.5	8.0	0.1	0.06	1.25	1.79	0.29
U1356A	15R	3W	20-22	137.0	13.44	0.42	5.4	4.9	0.1	0.06	1.18	1.83	0.02
U1356A	15R	5W	20-22	140.0	13.47	0.47	6.7	5.8	0.1	0.06	1.05	1.94	0.02
U1356A	17R	2W	20-22	154.7	13.61	0.50	15.1	12.1	0.1	0.09	1.21	1.81	0.22
U1356A	17R	4W	18-20	157.7	13.64	0.43	5.1	4.7	0.0	0.06	1.25	1.78	0.10
U1356A	18R	1W	18-20	162.7	13.69	0.45	10.8	8.9	0.1	0.10	1.30	1.74	0.17
U1356A	19R	1W	20-22	172.3	13.79	0.46	12.2	10.0	0.3	0.11	1.23	1.80	0.15
U1356A	20R	1W	19-21	181.9	13.88	0.45	9.2	7.7	0.1	0.07	1.13	1.88	0.04
U1356A	21R	1W	22-24	191.5	13.98	0.48	9.8	8.2	0.1	0.07	1.01	1.99	0.00
U1356A	22R	1W	22-24	201.1	14.07	0.45	12.6	10.3	0.1	0.08	1.14	1.86	0.05
U1356A	23R	1W	21-23	210.7	14.16				0.6	0.22	2.08	1.35	0.87
U1356A	24R	1W	21-23	220.3	14.25	0.43	8.5	7.2	0.1	0.07	1.14	1.87	0.01
U1356A	30R	3W	20-22	280.7	14.80				0.7	0.81	14.7	0.56	1.74
U1356A	30R	5W	19-21	283.7	14.86				0.7	0.23	0.06	3.28	-1.15
U1356A	33R	1W	29-31	306.7	15.26				0.8	0.17	5.90	0.62	1.29
U1356A	34R	2W	20-22	317.6	15.45				0.7	0.21	6.29	0.59	1.24
U1356A	35R	1W	21-23	325.7	15.59				0.4	0.24	3.08	1.05	0.95
U1356A	35R	3W	20-22	328.7	15.65				0.7	0.22	4.18	0.83	1.14
U1356A	39R	2W	20-22	365.3	16.29				0.8	0.18	2.86	1.09	0.98
U1356A	40R	1W	20-22	373.4	16.43				0.6	0.26	1.84	1.47	0.77
U1356A	41R	2W	20-22	384.5	16.63	0.52	17.4	13.9	0.1	0.15	0.99	2.00	0.10
U1356A	42R	1W	20-22	392.6	16.77	0.56	13.8	11.2	0.1	0.23	1.25	1.81	0.39
U1356A	42R	2W	39-43	394.1	16.80	0.55	20.1	16.0	0.2	0.20	1.56	1.61	0.56
U1356A	43R	1W	27-29	402.3	16.94	0.50	17.1	13.6	0.1	0.19	1.25	1.79	0.24
U1356A	43R	1W	118-122	403.2	16.95	0.55	21.0	16.6	0.2	0.23	1.25	1.81	0.37
U1356A	43R	2W	69-73	404.2	16.97				0.2	0.23	2.02	1.39	0.76

Supplementary Table 3 – TEX_{86}[11] values, Sea Surface Temperature (SST, 0 m)[12], Sea Water Temperature (0-200 m)[13], BIT (Branched and Isoprenoid Tetraether Index)[14] and MI (Methanogenic Index)[15, 16] for the studied Wilkes Land record (see Methods). In red are the values of the different indices based on which temperature values have been discarded (see Methods).

IODP Hole	Core	Sector	Depth (cm)	Depth (mbsf)	Age (Ma)	BIT index	MAT [17] (°C)	MAT [18] (°C)	MAT [19] (°C)
U1356A	19R	1W	20-22	172.3	13.79	0.3	7.1	9.1	-0.9
U1356A	23R	1W	21-23	210.7	14.16	0.6	8.5	11.0	5.9
U1356A	30R	3W	20-22	280.7	14.80	0.7	11.1	11.7	9.1
U1356A	30R	5W	19-21	283.7	14.86	0.7	10.2	*	*
U1356A	33R	1W	29-31	306.7	15.26	0.8	8.5	10.4	3.6
U1356A	34R	2W	20-22	317.6	15.45	0.7	11.4	12.2	10.9
U1356A	35R	1W	21-23	325.7	15.59	0.4	10.8	*	*
U1356A	35R	3W	20-22	328.7	15.65	0.7	9.7	11.8	13.7
U1356A	39R	2W	20-22	365.3	16.29	0.8	9.6	11.4	9.9
U1356A	40R	1W	20-22	373.4	16.43	0.6	8.3	11.5	8.6

Supplementary Table 4: annual mean air temperature (MAT) as calculated with different calibrations [17,18,19] for the Wilkes Land record. MAT has been calculated when BIT index ≥ 0.3[20] (see Methods). MAT[18] is that discussed in the main text.
[*] brGDGT concentration below detection limit;

Depth (mbsf)	Description	Type	Age (Ma)
255.2	*Actinocyclus ingens*	FAD	15.05-15.96
262.57	base cycle 60	Unconformity	15.2-15.8
263.0	absence *A. ingens*	FAD	>15.96
264.44	lower C5Br	reversed polarity	<15.974
328.52	C5Br/C5Cn.1n	MPR	15.974
358.11	lava clast (Diamictite)	$^{40}Ar/^{39}Ar$ date	16.01 +/- 0.17
366.8	*Adamusium* shell	$^{87}Sr/^{86}Sr$ date	16.05 +/- 0.35
412.71	C5Cn.1n/C5Cn.1r	MPR	16.268
429.56	C5Cn.1r/C5Cn.2n (?)	MPR	16.303
439.23	C5Cn.2n/C5Cn.2r (?)	MPR	16.472
448.59	C5Cn.2r/C5Cn.3n	MPR	16.543
482.69	C5Cn.3n/C5Cr (?)	MPR	16.721
564.92	lava clast (Conglomerate)	$^{40}Ar/^{39}Ar$ date	17.2+/-0.17
581.34	C5Cr/C5Dn	MPR	17.235

Supplementary Table 5: Tie points used to construct the age model for the early and mid-Miocene portion of the ANDRILL AND-2A record (Ross Sea, 77°45.49’S, 165°16.61’E, 380 m water depth). Age model detailed information are published in Levy et al.[21]

Depth (mbsf)	Age (Ma)	TEX_{86}	SST (0 m)	SWT (0-200 m)	BIT index	MI	GDGT 0/cren	Ring Index	ΔR
274.92	15.86	0.43	3.3	3.3	0.1	0.05	1.05	1.87	0.07
280.60	15.87	0.46	6.9	6.0	0.1	0.05	1.04	1.95	0.01
284.37	15.88	0.47	7.2	6.2	0.1	0.05	1.07	1.95	-0.02
285.35	15.88	0.45	7.1	6.2	0.1	0.05	1.05	1.91	0.04
292.52	15.89	0.45	6.7	5.9	0.1	0.05	1.01	1.92	0.06
295.25	15.90	0.44	2.9	3.0	0.1	0.05	1.04	1.89	0.07
299.42	15.91	0.44	3.1	3.1	0.2	0.05	1.14	1.90	-0.03
307.01	15.93	0.47	6.8	5.9	0.1	0.05	1.15	1.97	-0.11
310.24	15.93	0.44	7.6	6.6	0.1	0.06	1.01	1.89	0.10
311.07	15.94	0.44	7.2	6.2	0.1	0.05	1.03	1.88	0.08
312.75	15.94	0.46	2.6	2.8	0.0	0.05	0.89	1.93	0.18
316.57	15.95	0.46	2.2	2.5	0.2	0.06	1.15	1.93	-0.07
351.59	16.00	0.43	-0.6	0.4	0.0	0.03	0.71	1.87	0.46
426.29	16.29	0.45	6.5	5.7	0.2	0.05	1.01	1.92	0.07
431.63	16.33	0.45	7.6	6.5	0.1	0.06	1.01	1.91	0.08
433.49	16.35	0.44	8.2	7.0	0.1	0.05	1.05	1.90	0.04
437.20	16.40	0.42	2.3	2.5	0.3	0.06	1.35	1.84	-0.14
445.07	16.50	0.44	3.4	3.4	0.2	0.05	1.15	1.89	-0.04
455.27	16.58	0.44	5.7	5.1	0.3	0.05	1.18	1.90	-0.07
490.26	16.77	0.38	-1.4	-0.2	0.2	0.05	1.08	1.77	0.14
496.76	16.80	0.42	1.7	2.1	0.1	0.06	1.19	1.86	-0.04
501.02	16.83	0.44	1.2	1.7	0.3	0.05	1.08	1.89	0.03
513.31	16.90	0.46	3.1	3.1	0.3	0.05	1.04	1.95	0.01
526.28	16.97	0.46	6.6	5.8	0.1	0.06	1.12	1.94	0.20

Supplementary Table 6 – TEX_{86} values[11], Sea Surface Temperature (SST, 0 m)[12] Sea Water Temperature (0-200 m)[13], BIT (Branched and Isoprenoid Tetraether Index)[14] and MI (Methanogenic Index)[15,16] for the AND-2A record (see Methods)

Supplementary References

1. Tauxe, L. *et al.* Chronostratigraphic framework for the IODP Expedition 318 cores from the Wilkes Land Margin: constraints for paleoceanographic reconstruction. *Paleoceanography* **27**, PA2214 (2012).
2. Cody, R. *et al.* Selection and stability of quantitative stratigraphic age models: Plio-Pleistocene glaciomarine sediments in the ANDRILL 1B drillcore, McMurdo Ice Shelf. Global Planetary Change, **96-97,** 143-156 (2012)
3. Crampton, J. S. *et al.* Southern Ocean phytoplankton turnover in response to stepwise Antarctic cooling over the past 15 million years. *Proc. Natl. Acad. Sci. U. S. A.* **113**, 6868-6873 (2016).
4. Gradstein, F. M., Ogg, J. G. & Smith, A. G. in: *A geologic timescale 2004.* 589 Cambridge University Press, Cambridge (2004).
5. Escutia, C., Brinkhuis, H., Klaus, A. & Party, E. 3. S. Proceedings of the Integrated Ocean Drilling Program, Initial Results, volume 318. *Tokyo (Integrated Ocean Drilling Program Management International, Inc.)* (2011).
6. Prebble, J. G. *et al.* An expanded modern dinoflagellate cyst dataset for the Southwest Pacific and Southern Hemisphere with environmental associations. *Mar. Micropaleontol.* **101**, 33-48 (2013).
7. Raine, J. I., Mildenhall, D. C. & Kennedy, E. M. New Zealand fossil spores and pollen: an illustrated catalogue. *GNS Science miscellaneous series* **4** (2011).
8. Raine, J. I. Terrestrial Palynomorphs from Cape Roberts Project Drillhole CRP-1, Ross Sea, Antarctica. *Terra Antarct.* **5**, 539-548 (1998).
9. Raine, J. I. Zonate lycophyte spores from New Zealand Cretaceous to Paleogene strata. *Alcheringa* **32**, 99-127 (2008).
10. Truswell, E. M. & Macphail, M. K. Polar forests on the edge of extinction: what does the fossil spore and pollen evidence from East Antarctica say? *Australian systematic botany* **22**, 57-106 (2009).
11. Schouten, S., Hopmans, E. C., Schefuß, E. & Sinninghe Damsté, J. S. Distributional variations in marine crenarchaeotal membrane lipids: a new tool for reconstructing ancient sea water temperatures? *Earth Planet. Sci. Lett.* **204**, 265-274 (2002).
12. Kim, J. -. *et al.* New indices and calibrations derived from the distribution of crenarchaeal isoprenoid tetraether lipids: Implications for past sea surface temperature reconstructions. *Geochim. Cosmochim. Acta* **74**, 4639-4654 (2010).
13. Kim, J. -. *et al.* Holocene subsurface temperature variability in the eastern Antarctic continental margin. *Geophys. Res. Lett.* **39** (2012).
14. Hopmans, E. C. *et al.* A novel proxy for terrestrial organic matter in sediments based on branched and isoprenoid tetraether lipids. *Earth & Planetary Science Letters* **224**, 107-116 (2004).
15. Zhang, Y. G. *et al.* Methane Index: A tetraether archaeal lipid biomarker indicator for detecting the instability of marine gas hydrates. *Earth Plan. Sci. Lett.* **307**, 525-534 (2011).
16. Zhang, Y. G. *et al.* Ring Index: A new strategy to evaluate the integrity of TEX_{86} paleothermometry, *Paleoceanography* **31**, 220-232 (2016)
17. Peterse, F. *et al.* Revised calibration of the MBT-CBT paleotemperature proxy based on branched tetraether membrane lipids in surface soils. *Geochim. Cosmochim. Acta* **96,** 215-229 (2012).
18. De Jonge, C. *et al.* Occurrence and abundance of 6-methyl branched glycerol dialkyl glycerol tetraethers in soils: Implications for palaeoclimate reconstruction. Geochim. Cosmochim. Acta **141**, 97-112 (2014).

19 Naafs, B.D.A. *et al.* Refining the global branched glycerol dialkyl glycerol tetraether (brGDGT) soil temperature calibration. *Org. Geochem.* **106,** 476-491 (2017).

20. Weijers, J. W. H., Schouten, S., van den Donker, J. C., Hopmans, E. C. & Sinninghe Damsté, J. S. Environmental controls on bacterial tetraether membrane lipid distribution in soils. *Geochim. Cosmochim. Acta* **71**, 703-713 (2007).

21. Levy, R. *et al*. Antarctic ice sheet sensitivity to atmospheric CO_2 variations in the early to mid-Miocene. *Proc. Natl. Acad. Sci. U. S. A.* **113**, 3453-3458 (2016).
