# Supplementary material for: Southern Ocean warming and Wilkes Land ice sheet retreat during the mid-Miocene
Source: Nat Commun. 2018 Jan 22;9:317. doi: 10.1038/s41467-017-02609-7 (PMC5778126; doi:10.1038/s41467-017-02609-7)
Supplement: Supplementary file 2 — Description of Additional Supplementary Files [file 41467_2017_2609_MOESM2_ESM.pdf]

Description of Additional Supplementary Files

File Name: Supplementary Data 1
Description: Biostratigraphic datums using the Strict Hybrid CONOP model (Cody et al., 2012, ref. 2 Supplementary References).
This uses the methodology of Cody et al. 2012, but includes radiolarian addition to diatom data. The Composite Rank position represents the position within the CONOP composite sequence, while the observed depths of events (IODP U1356 (O)) are placed to revised depths (IODP U1356 (P)) using a iterative approach to correlated to a best fit to the composite sequence. The age model is this paper uses the placed depths of events observed in U1356A.

File Name: Supplementary Data 2
Description: Biostratigraphic datums using the Relaxed Hybrid CONOP model (Cody et al., 2012, ref 2 Supplementary References).
This uses the methodology of Cody et al. (2012), but includes radiolarian addition to diatom data. The Composite Rank position represents the position within the CONOP composite sequence, while the observed depths of events (IODP U1356 (O)) are placed to revised depths (IODP U1356 (P)) using a iterative approach to correlated to a best fit to the composite sequence. The age model is this paper uses the placed depths of events observed in U1356A.

File Name: Supplementary Data 3
Description: Lithological description of the cores. Lithological Units, depth sediments (mbsf), assigned ages, facies and interpretations.

File Name: Supplementary Data 4
Description: Dinoflagellate cyst relative and absolute abundances.

File Name: Supplementary Data 5
Description: Pollen and spore relative abundances.
